# Small Protein Hidden in lncRNA *LOC90024* Promotes “Cancerous” RNA Splicing and Tumorigenesis

**DOI:** 10.1002/advs.201903233

**Published:** 2020-03-11

**Authors:** Nan Meng, Min Chen, De Chen, Xin‐Hui Chen, Ji‐Zhong Wang, Song Zhu, Yu‐Tian He, Xiao‐Lan Zhang, Rui‐Xun Lu, Guang‐Rong Yan

**Affiliations:** ^1^ Biomedicine Research Center The Third Affiliated Hospital of Guangzhou Medical University Guangzhou 510150 China; ^2^ Guangzhou Municipal and Guangdong Provincial Key Laboratory of Protein Modification and Degradation State Key Laboratory of Respiratory Disease Guangzhou Medical University Guangzhou 511436 China

**Keywords:** colorectal cancer (CRC), lncRNA, prognostic biomarkers, RNA splicing, small proteins, transcription factors

## Abstract

Conventional therapies for late‐stage colorectal cancer (CRC) have limited effects because of chemoresistance, recurrence, and metastasis. The “hidden” proteins/peptides encoded by long noncoding RNAs (lncRNAs) may be a novel resource bank for therapeutic options for patients with cancer. Here, lncRNA *LOC90024* is discovered to encode a small 130‐amino acid protein that interacts with several splicing regulators, such as serine‐ and arginine‐rich splicing factor 3 (SRSF3), to regulate mRNA splicing, and the protein thus is named “Splicing Regulatory Small Protein” (SRSP). SRSP, but not *LOC90024* lncRNA itself, promotes CRC tumorigenesis and progression, while silencing of SRSP suppresses CRC tumorigenesis. Mechanistically, SRSP increases the binding of SRSF3 to exon 3 of transcription factor *Sp4*, resulting in the inclusion of *Sp4* exon 3 to induce the formation of the “cancerous” long Sp4 isoform (L‐Sp4 protein) and inhibit the formation of the “noncancerous” short Sp4 isoform (S‐Sp4 peptide), which lacks the transactivation domain. The upregulated SRSP level is positively associated with malignant phenotypes and poor prognosis in patients with CRC. Collectively, the findings uncover that a lncRNA‐encoded small protein SRSP induces “cancerous” Sp4 splicing variant formation and may be a potential prognostic biomarker and therapeutic target for patients with CRC.

## Graphical abstract

The “hidden” proteins/peptides encoded by long noncoding RNAs (lncRNAs) may be a novel resource bank for therapeutic options for patients with cancer. The lncRNA LOC90024 encodes a small 130‐amino acid protein “Splicing Regulatory Small Protein” that binds to splicing regulator, serine‐ and arginine‐rich splicing factor 3 to promote the “cancerous” RNA splicing of the transcription factor Sp4, thereby promoting tumorigenesis.

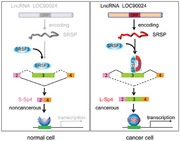

## 1 Introduction

Colorectal cancer (CRC) is one of the most common human malignancies worldwide and the incidence continues to rapidly increase in many low‐income and middle‐income countries.^[^ ^1^ ^]^ Patients with early stage CRC respond well to the current treatment with a high 5‐year survival rate, but the prognosis of patients with late‐stage diseases (especially stage IV) remains poor due to the high incidence of tumor recurrence and distant metastasis.^[^ ^2^ ^]^ Most patients with late‐stage CRC do not respond to current chemotherapies. Therefore, there is an urgent need to further understand the molecular mechanisms in CRC pathogenesis and to identify new therapeutic targets and prognostic biomarkers for CRC.

Transcription factors (TFs) represent the key point of convergence of multiple signaling pathways in cells. Deregulation of TFs results in the misregulation of gene expression and contributes to the pathogenesis of the majority of human diseases, including cancers.^[^ ^3^ ^]^ Thus, TFs hold great therapeutic potential. In cancer cells, genes that encode TFs are often amplified, deleted, rearranged, or mutated. Many oncogenic signaling pathways alter the functions of TFs to implement gene expression changes, thereby driving tumorigenesis and cancer progression.^[^ ^4^, ^5^ ^]^ The deregulation of TFs by modifications (such as phosphorylation), sublocalization, stabilization, binding, and transactivation is linked to tumorigenesis and cancer progression.^[^ ^4^, ^5^ ^]^ RNA alternative splicing (AS) is an essential process that yields structurally and functionally distinct mRNA and protein variants to expand the functional and regulatory capacity of metazoan genomes. Accumulating evidence indicates that aberrant AS controls various hallmarks of cancer.^[^ ^6^, ^7^, ^8^, ^9^ ^]^ However, the functional roles of the AS of TF RNAs in cancers remain poorly understood.

Approximately 2% of transcripts have protein‐coding potential. However, developments in deep‐sequencing technologies have led to the discovery of many previously unannotated nonprotein coding transcripts. Transcripts of more than 200 nucleotides that are not protein‐coding open reading frames (ORFs) are defined as long noncoding RNAs (lncRNAs). LncRNAs have been identified to play significant roles in cells. Consequently, dysregulation of lncRNAs in cancers contributes to the hallmarks of cancers and the survival time of cancer patients; thus, they are attractive potential biomarkers and therapeutic targets.^[^ ^8^, ^10^, ^11^ ^]^ However, the functional roles of lncRNA in “cancerous” RNA splicing remain to be explored.

In this study, we reveal that *LOC90024*, which is annotated as a lncRNA in *Homo sapiens*, encodes a small 130‐aa protein which we named “Splicing Regulatory Small Protein” (SRSP). The *LOC90024* and SRSP levels are frequently upregulated in CRC tissues. CRC patients with high SRSP levels exhibit more aggressive clinicopathological phenotypes and shorter survival times than those with low levels of SRSP. SRSP, but not its *LOC90024* lncRNA itself, promotes CRC tumorigenesis and progression in vitro and in vivo. SRSP interacts with splicing factor serine‐ and arginine‐rich splicing factor 3 (SRSF3) to increase the binding of SRSF3 to exon 3 of transcription factor Sp4 and resulted in the inclusion of exon 3, thereby inducing formation of the long Sp4 isoform (L‐Sp4 protein) and suppressing formation of the short Sp4 isoform (S‐Sp4 peptide) which has no transactivation domain to promote CRC tumorigenesis and progression. The blockade of the binding of SRSP to SRSF3 inhibits L‐Sp4 formation, CRC tumorigenesis, and progression. Thus, we reveal that lncRNA *LOC90024* encodes a small protein SRSP that regulates Sp4 splicing and CRC tumorigenesis and propose SRSP as a novel prognostic biomarker and therapeutic target of CRC.

## 2 Results

### 2.1 LncRNA *LOC90024* Encodes a Small Protein

**Figure 1:**
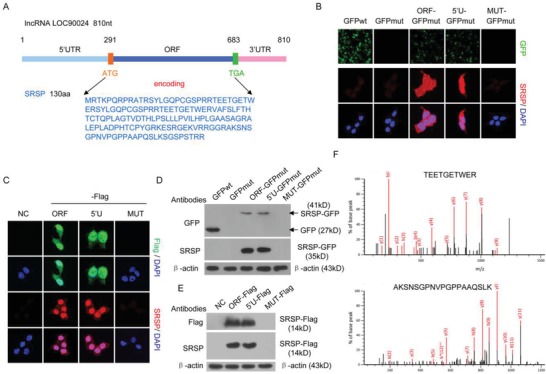
LncRNA *LOC90024* encodes a 130‐aa small protein, SRSP. A) 840 nt lncRNA *LOC90024* contains a potential small protein‐encoding ORF, which may encode a 130‐aa small protein, SRSP. B,D) The *LOC90024* ORF, 5′UTR‐ORF (5′U), or 5′UTR‐ORFmut (MUT, in which the start codon ATG of SRSP ORF was mutated to ATT) constructs fused with *GFP*, in which the start codon of *GFP* was mutated (GFPmut), were transfected into HeLa cells; B) GFP fluorescence and SRSP‐GFP immunostaining were detected using anti‐SRSP antibody and D) SRSP‐GFP fusion protein expression was detected by Western blotting with anti‐GFP and SRSP antibodies. C,E) The *LOC90024* ORF, 5′U, or MUT constructs fused with a *Flag* tag were transfected into HeLa cells, and C) SRSP‐Flag immunostaining and E) expression were detected using anti‐Flag and ‐SRSP antibodies. F) Two unique peptides in the ectopically expressed SRSP‐GFP were identified using mass spectrometry.

To discover the potential lncRNAs which can encode proteins/peptides, we used the purification of ribosome‐bound RNAs with RNA‐sequencing to identify the ribosome‐bound lncRNAs (GSE139407). Our ribosome‐bound RNA‐seq analysis showed that lncRNA *LOC90024* could bind to ribosome, suggesting that either *LOC90024* may be translated to protein/peptide or may participate in the regulation of translation as a lncRNA molecule. *LncRNA LOC90024* was annotated as a lncRNA with unknown functions in *H. sapiens* (NR_040415). Our bioinformatics analysis showed that there is a 393‐nucleotide ORF with the potential to encode a 130‐amino acid small protein in *LOC90024* (**Figure** **1**A). We named this small protein SRSP. Homology assays showed that SRSP was highly conserved in primates, including humans, but there were no classical domains/motifs in SRSP, suggesting that SRSP is an uncharacterized, novel protein with unknown functions.

To confirm that the predicted ORF in lncRNA *LOC90024* can be translated, the start codon ATG within the predicted ORF was mutated to ATT. Mutation of the start codon ATG of the predicted ORF in *LOC90024* abolished the expression of the SRSP‐GFP or SRSP‐Flag fusion protein (Figure 1B–E). To further investigate the translation of the ORF of SRSP in *LOC90024*, we generated an antibody specific to SRSP and verified its specificity (Figure S1, Supporting Information). Similarly, the expression of the SRSP‐GFP or SRSP‐Flag fusion protein was not detected by the anti‐SRSP antibody when the start codon ATG of the predicted ORF in *LOC90024* was mutated (Figure 1B–E). Finally, the SRSP‐GFP fusion protein was further identified by mass spectrometry (Figure 1F). Collectively, these data indicate that lncRNA *LOC90024* actually encodes a small protein SRSP.

### 2.2 SRSP Is Naturally, Endogenously Produced in Human Cells and Tissues

**Figure 2:**
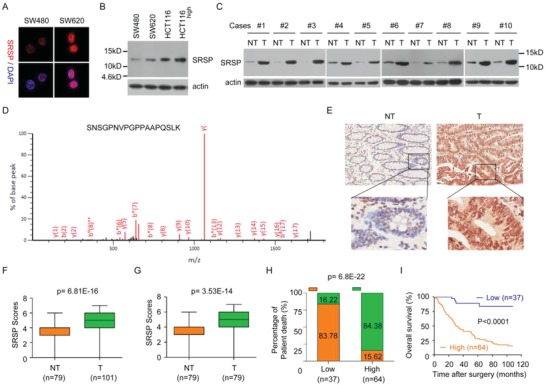
The *LOC90024*‐encoded SRSP is endogenously, naturally expressed, and SRSP is upregulated in cancers and associated with the prognosis of patients with CRC. A) SRSP immunostaining between SW480 and SW620 cells was determined with an anti‐SRSP antibody. B) SRSP levels were increased in high‐metastatic CRC cells compared with their parental low‐metastatic CRC cells. C) SRSP levels were increased in tumor tissues (T) compared with their matched adjacent nontumoral tissues (NT). D) The unique peptide in the endogenous, natural SRSP in tumor tissues was identified using mass spectrometry. E) Representative IHC images of SRSP expression in CRC T and their matched NT. F) Differences in SRSP scores between T and NT are presented as a box plot. G) Differences in SRSP scores between T and their matched NT are presented as a box plot. H) CRC patients with high SRSP levels had a higher death rate than those with low SRSP levels. I) CRC patients with high SRSP levels had shorter survival times than those with low SRSP levels.

To validate the presence and expression of the natural, endogenous SRSP, SRSP was detected in cells and tissues using the anti‐SRSP antibody and mass spectrometry. We found that natural, endogenous SRSP was present in several cancer cell types, such as colorectal, breast, ovarian, and nasopharyngeal cancer cells (**Figure** **2**A,B and Figure S2A, Supporting Information). Furthermore, the presence of the natural, endogenous SRSP was also verified in fresh primary cancer tissues and their corresponding adjacent nontumoral tissues (Figure 2C). More importantly, the naturally endogenously produced SRSP in cancer tissues was identified and validated by mass spectrometry (Figure 2D). Taken together, these data suggest that SRSP is endogenously, naturally produced in human cells and tissues.

### 2.3 *LOC90024* and SRSP Levels Are Increased in CRC and Its Upregulation Is Associated with Poor Prognosis in Patients with CRC

Our ribosome‐bound RNA‐seq data showed that *LOC90024* levels were increased in SW620 cells compared to SW480 cells. The upregulation of *LOC90024* expression was further confirmed in highly metastatic colorectal, ovarian, nasopharyngeal, and breast cancer cell sublines compared with their parental cell lines (Figure S2B,C, Supporting Information). Similarly, SRSP levels were also substantially upregulated in these highly metastatic cancer cell sublines compared with their parental cell lines (Figure 2B and Figure S2A, Supporting Information).

The *LOC90024* and SRSP expression levels were further determined in ten pairs of fresh primary cancer tissues and their matched nontumoral tissues. *LOC90024* and SRSP levels were substantially higher in cancer tissues than in their matched nontumoral tissues (Figure 2C and Figure S2D,E, Supporting Information). Unfortunately, no *LOC90024* expression data were obtained from the publicly accessible databases Cancer Genome Atlas (TCGA) and Oncomine.

The SRSP expression levels in 101 CRC tissue samples and 79 nontumoral colorectal tissue samples (including 79 pairs of CRC tissues and their matched nontumoral colorectal tissues) were further investigated using an immunohistochemistry (IHC) assay (Figure 2E). SRSP expression levels were significantly higher in CRC tissues than in adjacent nontumoral colorectal tissues (Figure 2F,G). Taken together, *LOC90024* and SRSP levels were substantially upregulated in CRC.

Furthermore, the correlations of SRSP levels with clinicopathological features of patients with CRC were investigated in 101 CRC cases. The upregulation of SRSP was positively associated with histological grade (*P* = 0.001), pN status (*P* = 0.026), and clinical stage (*P* = 0.004) of CRC (Table S1, Supporting Information). CRC patients with high SRSP levels had a higher risk of cancer death than those with low SRSP levels (Figure 2H). SRSP levels correlated significantly with the overall patient survival rate (*P* < 0.0001, log‐rank test) (Figure 2I). The median survival time for patients with CRC and high SRSP levels was 30 months, while that for patients with CRC and low SRSP levels was 100 months. A high SRSP level is an independent prognostic factor for poor survival of patients with CRC (hazard ratio (HR) = 7.692, 95% confidence interval (CI) = 2.982–19.841, *P* = 0.0001) (Table S2, Supporting Information). Collectively, these results reveal that SRSP is upregulated in CRC and its upregulation is significantly correlated with poor prognosis for patients with CRC, indicating that SRSP may play an oncogenic role in CRC.

### 2.4 SRSP, but Not *LOC90024* lncRNA Itself, Promotes CRC Tumorigenesis

To investigate the functions of *LOC90024* and SRSP in CRC tumorigenesis, *LOC90024*‐knockout (KO) cells were constructed by using CRISPR‐Cas9 technology (Figure S3A, Supporting Information). KO of *LOC90024* significantly suppressed cancer cell proliferation, colony formation, migration, and invasion (Figure S3B–E, Supporting Information). Similar results were obtained when *LOC90024* expression was knocked down (KD) by two anti‐*LOC90024* siRNAs in HCT‐116 and SW620 CRC cells (Figure S3F–H, Supporting Information). These data indicate that KO or KD of the *LOC90024* gene inhibits CRC tumorigenesis. However, we did not know whether the *LOC90024* lncRNA or the LOC90024‐encoded SRSP is at work.

**Figure 3:**
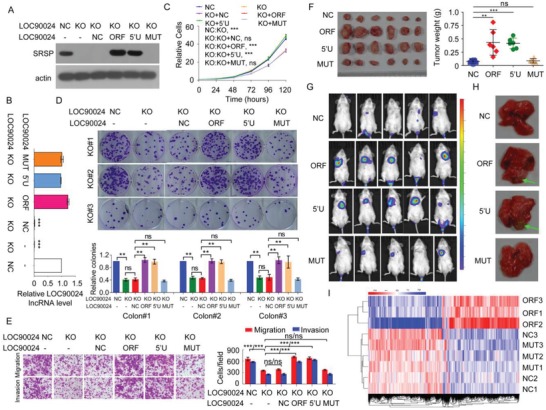
SRSP, but not *LOC90024* lncRNA itself, stimulates CRC tumorigenesis and metastasis in vitro and in vivo. A–D) The indicated *LOC90024* constructs were transfected into *LOC90024* KO HeLa cells, A) SRSP expression, B) LOC90024 lncRNA level, C) cell growth, D) colony formation, E) migration and invasion were determined. F) The in vivo tumorigenesis of the indicated HCT‐116 cells stably expressing the indicated *LOC90024* constructs was determined. The images and weights of the xenograft tumors are listed in the left and right panels, respectively (*n* = 6). G,H) The indicated Luc‐labeled HCT‐116 cells stably expressing the indicated *LOC90024* constructs (2 × 10^6^ cells per mouse) were injected into NOD‐SCID mice via the tail vein; G) luciferase activities and H) metastases in mouse lung were visualized 50 d posttransplantation (*n* = 5). I) The cluster heatmap of gene expression in negative control (NC), *LOC90024 ORF* (*ORF*), and *5′UTR‐ORFmut* (*MUT*) overexpression (*n* = 3). Data are represented as mean ± SD. **p < 0.01 or ***p < 0.001.

To further investigate and differentiate the functions of SRSP and its *LOC90024* lncRNA in CRC tumorigenesis, we restored expression with *LOC90024* ORF‐Flag, 5′UTR‐ORF‐Flag (5′U), or 5′UTR‐ORFmut‐Flag (MUT) vectors in *LOC90024* KO cells. We found that the *LOC90024* KO‐induced alterations were restored to the control level after reexpression with *LOC90024* ORF‐Flag and 5′UTR‐ORF‐Flag (5′U), which could encode SRSP, but not after the reexpression of *LOC90024* 5′UTR‐ORFmut‐Flag, which was a lncRNA that did not encode SRSP (**Figure** **3**A–E). In addition, we transfected the *LOC90024* ORF, 5′UTR‐ORF, and 5′UTR‐ORFmut Flag fusion constructs into SW480 and HCT‐116 CRC cells. Similarly, the *LOC90024* ORF and 5′UTR‐ORF constructs promoted cancer cell proliferation, colony formation, migration, and invasion, whereas the *LOC90024* 5′UTR‐ORFmut construct did not change CRC cell proliferation, colony formation, migration, and invasion (Figure S4, Supporting Information).

The tumors that arose in mice injected with CRC cells with *LOC90024* ORF‐Flag or 5′UTR‐ORF‐Flag stable expression were much larger than those in mice that were injected with CRC cells stably expressing blank vector (NC) or 5′UTR‐ORFmut‐Flag; the in vivo xenograft tumor growth with 5′UTR‐ORFmut‐Flag expression was similar to that with blank vector expression (Figure 3F). In addition, the metastatic nodules that developed in mice after tail vein injections with luciferase‐tagged HCT‐116 CRC cells stably expressing *LOC90024* ORF‐Flag or 5′UTR‐ORF‐Flag were larger than the nodules in mice injected with cells stably expressing blank vector or *LOC90024* 5′UTR‐ORFmut‐Flag; the metastatic nodules in the lungs of mice with 5′UTR‐ORFmut‐Flag expression was similar to that in mice with blank vector expression (Figure 3G,H).

Furthermore, we used RNA‐seq to measure the transcriptome alterations mediated by *LOC90024* and SRSP. The mRNA levels of 2816 genes were altered (upregulated and downregulated) by SRSP overexpression, while the mRNA levels of only 43 genes were altered by noncoding *LOC90024* lncRNA overexpression (*p* < 0.01) (GSE139406). The transcriptome profiles mediated by *LOC90024* overexpression and negative control were clustered into a group, different from those mediated by SRSP overexpression (Figure 3I), and this was consistent with the functional effects of SRSP, which promotes tumorigenesis while *LOC90024* lncRNA does not. Taken together, these data indicate that SRSP, not *LOC90024* lncRNA itself, promotes tumorigenesis.

### 2.5 SRSP Interacts with Splicing Factor SRSF3

**Figure 4:**
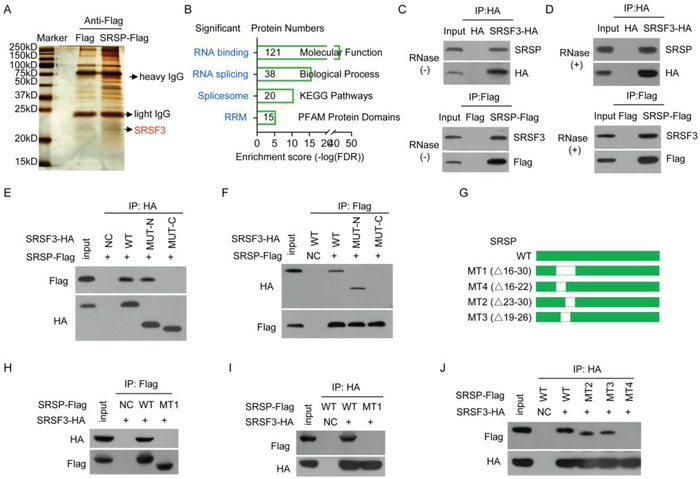
SRSP interacts with the splicing factor SRSF3. A) Proteins that interacted with SRSP were identified by interactomics analysis. B) The SRSP‐bound proteins were analyzed by GO. C,D) The *SRSF3‐HA* or *SRSP‐Flag* plasmids were transfected into HEK293T cells, cellular lysates were treated D) with or C) without RNase A; SRSF3‐HA or SRSP‐Flag complexes were co‐IPed with anti‐HA or Flag antibodies, and then the indicated proteins were detected. E,F) The wild type (WT), N‐terminal (MUT‐N), and C‐terminal (MUT‐C) mutants of *SRSF3‐HA* together with *SRSP‐Flag* vector were cotransfected into HEK293T cells, and E) SRSF3‐HA and F) SRSP‐Flag complexes were co‐IPed with anti‐HA and ‐Flag antibodies, respectively, to detect SRSP‐Flag and SRSF3‐HA. G) Diagram of wild‐type *SRSP* and its mutant constructs. H–J) The indicated *SRSP‐Flag* mutants together with the *SRSF3‐HA* vector were cotransfected into HEK293T cells; the interaction of the SRSP mutant with SRSF3 was determined.

To unveil the mechanism by which SRSP promotes tumorigenesis and cancer progression, we searched for interacting partners of SRSP by interactomics analysis. A total of 574 SRSP‐interacting proteins were identified by mass spectrometry (**Figure** **4**A and Table S3, Supporting Information). Gene ontology (GO) analyses showed that these SRSP‐interacting proteins were mainly classified as RNA binding proteins (*P* = 3.69 × 10^−44^), significantly enriched in RNA splicing (*P* = 2.39 × 10^−16^) and involved in spliceosome pathway (*P* = 3.20 × 10^−11^), and they also contained RNA recognition motifs (RRMs) (*P* = 4.40 × 10^−6^), suggesting that SRSP may mainly regulates RNA splicing by interacting with splicing regulators (Figure 4B). Therefore, we named this *LOC90024*‐encoded small protein SRSP.

Among the 38 potential SRSP‐interacting splicing regulators, the interaction with SRSF3 was particularly interesting and was selected for further investigation because SRSF3 is a major splicing factor in AS and has critical roles in the regulation of the premRNA splicing, and dysregulation of SRSF3 contributes to tumorigenesis and progression of several human cancers.^[^ ^12^, ^13^ ^]^ We further confirmed that SRSP could interact with SRSF3 (Figure 4C). SRSF3 is an RNA splicing factor and RNA‐binding protein. Therefore, we further investigated whether SRSP interacts with SRSF3 through RNA. In the presence of RNase treatment, SRSP still interacted with SRSF3 (Figure 4D), indicating that the interaction of SRSP with SRSF3 does not depend on RNA.

SRSF family proteins contain an RRM at the N‐terminus for binding to RNA and an RS domain at the C‐terminus for binding to other partner proteins.^[^ ^13^ ^]^ To dissect the domain of SRSF3 required for interaction with SRSP, N‐terminus (1–90 aa) (MUT‐N) and C‐terminus (91–164 aa) (MUT‐C) mutants of *SRSF3‐hemagglutinin (HA)* were constructed and coexpressed in HEK293T cells together with the *SRSP‐Flag* vector. Only SRSF3 containing the N‐terminus could interact with SRSP, which is inconsistent with previous reports in which the C‐terminus of SRSF3 interacts with other partner proteins (Figure 4E,F). Therefore, the N‐terminus instead of the classic C‐terminus of SRSF3 is responsible for its interaction with SRSP.

SRSP is an uncharacterized, novel small protein without a known domain or motif. To determine which regions or residues of SRSP interact with SRSF3, a series of SRSP‐Flag mutants were constructed (Figure 4G and Figure S5A, Supporting Information) and coexpressed in HEK293T cells with SRSF3‐HA. We demonstrated that the N‐terminus of SRSP (1–60 aa) interacts with SRSF3 (Figure S5B, Supporting Information). Furthermore, we showed that only SRSP containing the 16–30 aa regions, but not other regions, could interact with SRSF3 (Figure 4H,I and Figure S5C, Supporting Information), and the proline‐arginine‐arginine (PRR) motif in the region was not essential for the interaction (Figure S5D, Supporting Information). Finally, we confirmed that the 16–22 aa region of SRSP interacted with SRSF3 (Figure 4J). Therefore, the GQPCGSP amino acids (the 16–22 aa region) in SRSP are responsible for its interactions with the N‐terminus of SRSF3.

### 2.6 SRSP Stimulates CRC Tumorigenesis via SRSF3

As expected, KD of *SRSF3* inhibited HCT‐116 and SW620 CRC cell growth, colony formation, migration, and invasion (Figure S6, Supporting Information), similar to the cancer phenotypes mediated by *SRSP* KD or KO. To determine whether SRSP stimulates CRC tumorigenesis via SRSF3, HCT‐116, and SW480 CRC cells were cotransfected with *SRSP‐Flag* and anti‐*SRSF3* siRNA. KD of *SRSF3* completely attenuated the enhancement in CRC cell growth, colony formation, migration, and invasion mediated by *SRSP‐Flag* overexpression (Figure S7, Supporting Information), indicating that SRSP stimulates CRC tumorigenesis mainly via SRSF3.

### 2.7 SRSP Strengthens the Binding of SRSF3 to *Sp4* Exon 3 and the Inclusion of *Sp4* Exon 3

We used RNA‐seq to measure the AS events of mRNAs potentially mediated by SRSP in CRC cells (GSE139406), considering that SRSP interacts with splicing regulators such as SRSF3, which is a critical splicing factor, exerts its functions by regulating RNA splicing. SRSP overexpression promoted 3792 splicing events including 311 alternative 3′ splice site (A3SS), 234 alternative 5′ splice site (A5SS), 633 mutually exclusive exon, 237 retained intron, and 2377 skipped exon events (false discovery rate (FDR) < 0.01), indicating that SRSP plays an important role in pre‐mRNA splicing by interacting with splicing regulators such as SRSF3.

**Figure 5:**
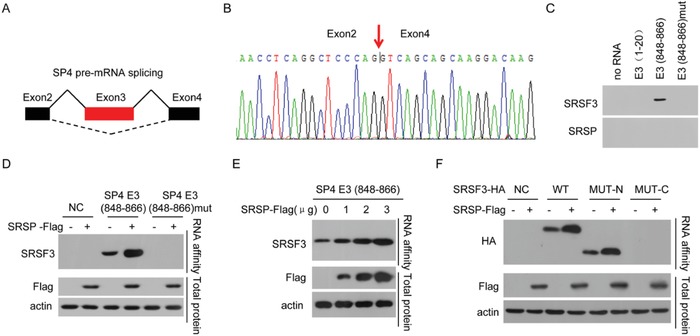
SRSP promotes the binding of SRSF3 to exon 3 of *Sp4*. A,B) SRSP promotes the inclusion of *Sp4* exon 3 (E3) using RNA‐seq analysis. C) The cellular nuclear extracts were affinity‐purified using the indicated biotin‐labeled RNAs, and SRSF3 and SRSP in purified complexes were detected. D) HCT‐116 cells were transfected with the *SRSP‐Flag* vector, RNA affinity purification was performed using the indicated biotin‐labeled RNAs, and SRSF3 was detected. E) *SRSP‐Flag* vector at the indicated doses was transfected into HCT‐116 cells, RNA affinity purification was performed using biotin‐labeled RNA E3 (848–866), and SRSF3 was detected. F) HCT‐116 cells were cotransfected with the indicated *SRSF3‐HA* mutants together with the *SRSP‐Flag* vector, RNA affinity purification was performed using biotin‐labeled RNA E3 (848–866), and SRSF3‐HA was detected.

TFs represent the key point of convergence of multiple signaling pathways in cells and play critical roles in tumorigenesis and cancer progression because TFs can regulate comprehensive transcription alterations in cancer cells.^[^ ^3^, ^4^, ^5^ ^]^ Sp4 is a general TF that binds to and acts through GC base boxes, which are frequently occurring DNA elements present in many promoters and enhancers.^[^ ^14^ ^]^ We found that SRSP promoted the inclusion of exon 3 of TF *Sp4* to promote long *Sp4* splicing variant formation (we termed L‐Sp4) and suppress short *Sp4* splicing variant formation without exon 3 (we termed S‐Sp4) (**Figure** **5**A). The skipping of exon 3 of *Sp4* was confirmed by targeted RNA‐seq when SRSP expression was knocked out (Figure 5B).

The SRSF3‐binding motif sequence CAACCA was found in *Sp4* exon 3. To confirm that SRSF3 can bind to *Sp4* exon 3, we prepared a 5′‐biotin‐labeled RNA spanning the predicted SRSF3‐binding motif, CAACCA, in *Sp4* exon 3 (E3(848–866)), a 5′‐biotin‐labeled RNA E3(848–866)mut in which the CAACCA was mutated to GTTGGT, and a 5′‐biotin‐labeled negative control RNA E3(1–20) corresponding to the exon 3 region 1–20. We performed RNA affinity chromatography using these 5′‐biotin‐labeled RNA probes and found that SRSF3 strongly bound to the *Sp4* E3(848–866) RNA probe but not the negative control *Sp4* E3(1–20) RNA probe (Figure 5C). The mutation of SRSF3‐bound sites completely abrogated the binding of SRSF3 to the *Sp4* E3(848–866) RNA probe. However, SRSP itself did not directly bind to these *Sp4* exon 3 RNA probes, indicating that SRSP does not directly recognize and bind to *Sp4* exon 3 (Figure 5C). Taken together, our data indicate that SRSF3 binds to *Sp4* exon 3 but SRSP does not.

The influence of SRSP on the binding of SRSF3 to *Sp4* exon 3 was further investigated. We found that *SRSP* overexpression markedly enhanced the binding of SRSF3 to the E3(848–866) of *Sp4* but did not induce the binding of SRSF3 to *Sp4* exon 3 when the SRSF3‐bound site was mutated in exon 3 of *Sp4* (Figure 5D). In addition, the binding of SRSF3 to *Sp4* exon 3 was increased in an SRSP‐dose‐dependent manner (Figure 5E).

SRSF3 contains an RRM domain at the N‐terminus for binding to RNA and an RS domain at the C‐terminus for binding to other partner proteins.^[^ ^13^ ^]^ As expected, the N‐terminus of SRSF3 recognized and bound to the E3(848–866) of *Sp4* (Figure 5F). Furthermore, SRSP, which also bound to the N‐terminus of SRSF3, strengthened the binding of the N‐terminus of SRSF3 to the E3(848–866) of *Sp4* (Figure 5F). Collectively, SRSP increased the binding and recognition of exon 3 of *Sp4* by SRSF3.

### 2.8 SRSP Drives SRSF3‐Dependent Inclusion of Exon 3 of *Sp4*

**Figure 6:**
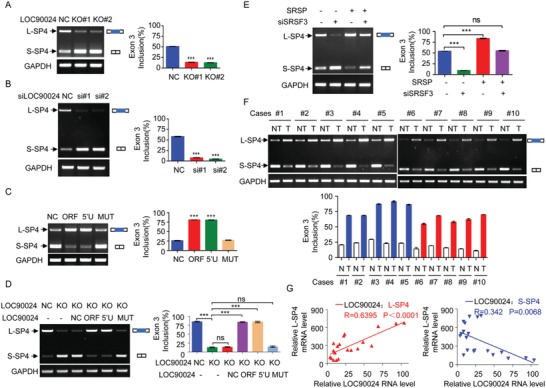
SRSP promotes the inclusion of exon 3 of *Sp4*. A) L‐Sp4 and S‐Sp4 splicing variants were determined by RT‐PCR in *LOC90024* KO HeLa cells. B) HCT‐116 cells were transfected with two anti‐*LOC90024* siRNAs, and levels of L‐Sp4 and S‐Sp4 splicing variants were determined. C) The indicated *LOC90024* constructs were transfected into HCT‐116 cells, and levels of L‐Sp4 and S‐Sp4 splicing variants were determined. D) The indicated *LOC90024* constructs were transfected into *LOC90024*‐KO HeLa cells, and levels of L‐Sp4 and S‐Sp4 splicing variants were determined. E) HCT‐116 cells were transfected with *SRSP* vector together with anti‐*SRSF3* siRNA, and levels of L‐Sp4 and S‐Sp4 splicing variants were determined. F) Levels of L‐Sp4 and S‐Sp4 splicing variants were determined between T and their matched NT. G) The relationships of *LOC90024* lncRNA levels with L‐Sp4 (upper panel) and S‐Sp4 (low panel) mRNA levels were analyzed in clinical tissue samples. Data are represented as mean ± SD. ***p < 0.001.

Given that SRSP enhances the binding and recognition of exon 3 of *Sp4* by SRSF3, the influences of SRSP on *Sp4* premRNA splicing were further investigated. KO of *LOC90024* impaired the inclusion of exon 3 of *Sp4* to decrease L‐Sp4 formation and enhance S‐Sp4 formation (**Figure** **6**A). Similar results were obtained in *LOC90024*‐KD cells (Figure 6B). The overexpression of *LOC90024* ORF and 5′UTR‐ORF (5′U) (encoding SRSP) increased L‐Sp4 formation and decreased S‐Sp4 formation, while the overexpression of *LOC90024* 5′UTR‐ORFmut (MUT) (not encoding SRSP) did not change *Sp4* premRNA splicing (Figure 6C). We restored the expression of *LOC90024* with *LOC90024* ORF, 5′UTR‐ORF (5′U), or 5′UTR‐ORFmut (MUT) vectors in *LOC90024* KO cells. The *LOC90024* KO‐impaired inclusion of *Sp4* exon 3 was restored to the control level after the reexpression of SRSP, but not *LOC90024* lncRNA (Figure 6D). In addition, SRSP did not change the total *Sp4* mRNA level (Figure S8A–E). KD of *SRSF3* attenuated the increased inclusion of exon 3 of Sp4 induced by *SRSP* overexpression (Figure 6E).

Finally, *Sp4* pre‐mRNA splicing was investigated in clinical tumor tissue samples. The L‐Sp4 mRNA levels were increased and S‐Sp4 mRNA levels were reduced in tumor tissues compared to their matched adjacent nontumoral tissues (Figure 6F). The total *Sp4* mRNA levels between tumor tissues and their matched adjacent nontumoral tissues were not different (Figure S8F, Supporting Information). The L‐Sp4 levels were positively correlated with SRSP levels in clinical tissue samples, while the S‐Sp4 levels were negatively correlated with SRSP levels (Figure 6G). Collectively, SRSP promotes SRSF3‐dependent inclusion of *Sp4* exon 3 to induce L‐Sp4 formation and suppress S‐Sp4 formation.

### 2.9 L‐Sp4, Not S‐Sp4, Stimulates CRC Tumorigenesis

**Figure 7:**
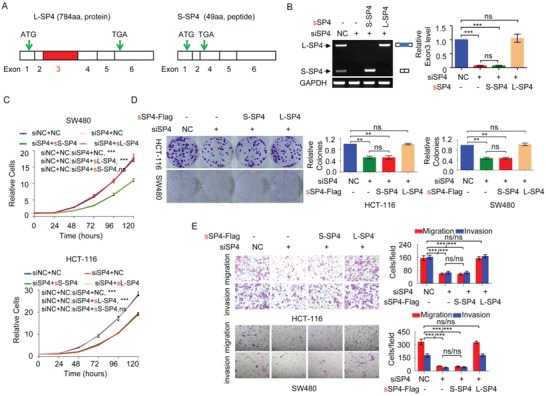
The L‐Sp4 isoform exerts an oncogenic function, but the S‐Sp4 isoform does not. A) Schematic diagram of the L‐Sp4 and S‐Sp4 splicing variants, which encode a 784 aa protein and a 49 aa peptide without the transactivation domain of Sp4, respectively. B–E) CRC cells were cotransfected with anti‐*Sp4* siRNA together with synonymously mutated *S‐Sp4* or *L‐Sp4* vector, which was resistant to anti‐*Sp4* siRNA. B) The L‐Sp4 and S‐Sp4 splicing variant levels, C) cell growth, D) colony formation, and E) migration and invasion of these cells were determined. Data are represented as mean ± SD. **p < 0.01 or ***p < 0.001.

The L‐Sp4 splicing variant encodes the 784‐aa protein L‐Sp4 isoform, while the S‐Sp4 splicing variant without exon 3 encodes the 49‐aa peptide S‐Sp4 isoform due to a frame shift (**Figure** **7**A). To determine the functions of L‐Sp4 and S‐Sp4 in CRC tumorigenesis, we restored the expression of *L‐Sp4* or *S‐Sp4*, which is resistant to anti‐*Sp4* siRNA, in *Sp4*‐KD CRC cells (Figure 7B). KD of *Sp4* inhibited CRC cell growth, colony formation, migration, and invasion in HCT‐116 and SW480 cells. The reduction of cell growth, colony formation, migration, and invasion induced by KD of *Sp4* in HCT‐116 and SW480 CRC cells could be efficiently rescued to the control level after the reexpression of *L‐Sp4* but not *S‐Sp4* (Figure 7C–E), indicating that the L‐Sp4 isoform exerts oncogenic functions, while the S‐Sp4 isoform has no oncogenic functions.

### 2.10 SRSP Stimulates CRC Tumorigenesis Mainly through L‐Sp4

Next, we investigated whether SRSP stimulates CRC tumorigenesis mainly through L‐Sp4, considering that SRSP promotes SRSF3‐dependent L‐Sp4 formation. CRC HCT‐116 and SW620 cells were cotransfected with the *SRSP‐Flag* construct together with anti‐L‐*Sp4* siRNA (Figure S9A, Supporting Information). The enhancement of cell growth, colony formation, and migration and invasion mediated by *SRSP* overexpression was completely attenuated by KD of *L‐Sp4* (Figure S9B–D, Supporting Information).

Furthermore, we downloaded and analyzed a publically accessible database about gene changes mediated by SP4 in *Sp4*‐depleted mouse cerebellum (GSE53061),^[^ ^15^ ^]^ in which the expression of 161 genes was decreased in expression in *Sp4* hypomorph cerebellum compared to wild type (WT, cutoff of *p* < 0.05 and fold change >1.5). We found that the expression of 2308 genes was upregulated by SRSP overexpression (cutoff of *p* < 0.05). The 93 of 161 expression‐altered genes mediated by Sp4 were also altered by SRSP. We randomly selected the ten downstream shared target genes for SRSP and Sp4 for further validation using qRT‐PCR. The expressions of all ten selected genes were decreased by both *Sp4* knockdown and *SRSP* knockout (Figure S9E,F, Supporting Information). Collectively, SRSP stimulates CRC tumorigenesis mainly through L‐Sp4.

### 2.11 SRSP Promotes *Sp4* Splicing and Tumorigenesis through Its Interaction with SRSF3

**Figure 8:**
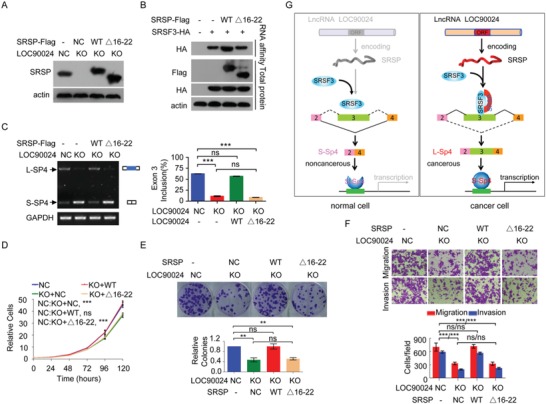
SRSP promotes the binding of SRSF3 to *Sp4* exon 3, which facilitates exon 3 inclusion in *Sp4* and tumorigenesis mainly by interacting with SRSF3. The wild‐type *SRSP* and its mutant Δ16–22 were re‐expressed in *LOC90024* KO HeLa cells; A) SRSP protein level, B) the binding of SRSF3 to *Sp4* exon 3, C) *Sp4* splicing, D) cell growth, E) colony formation, and F) migration and invasion were analyzed. G) A regulatory model of *LOC90024*‐encoded small protein SRSP on tumorigenesis proposed in this study. Data are represented as mean ± SD. **p < 0.01 or ***p < 0.001.

To investigate whether SRSP promotes the binding of SRSF3 to *Sp4* exon 3, the inclusion of *Sp4* exon 3, and the tumorigenesis of cells through its interaction with SRSF3, the expression of the wild‐type *SRSP* or the SRSF3 binding‐defective *SRSP* Δ16–22 mutant was restored in *LOC90024* KO cells (**Figure** **8**A). We found that SRSP did not increase the binding of SRSF3 to the E3(848–866) of *Sp4* when the binding region of SRSP to SRSF3 was deleted in SRSP (Figure 8B). SRSP did not induce the inclusion of exon 3 of *Sp4* to promote the L‐Sp4 splicing variant formation when the binding region of SRSP to SRSF3 in SRSP was deleted (Figure 8C). Functionally, the reexpression of wild‐type SRSP efficiently restored the reductions in cell growth, colony formation, migration, and invasion induced by KO of *LOC90024* to the control levels, while the SRSF3 binding‐defective SRSP Δ16–22 mutant did not (Figure 8D–G). Thus, these results indicate that SRSP promotes *Sp4* splicing and tumorigenesis mainly by its interaction with SRSF3.

## 3 Discussion

As one of the most prevalent methods of gene regulation, AS of RNA is an essential process that yields transcriptomic and proteomic diversity in eukaryotic cells. Aberrant splicing is closely associated with numerous human diseases, such as cancers, and contributes to all the hallmarks of cancer.^[^ ^6^, ^7^ ^]^ AS is generally orchestrated by *cis*‐RNA elements that recruit splicing factors to enhance or silence the splicing of adjacent exons. Common splicing factors include serine/arginine‐rich proteins and heterogeneous nuclear ribonucleoproteins (hnRNPs).^[^ ^6^, ^8^ ^]^ The expression level dysregulation, activity, post‐translational modification, and subcellular localization of these splicing factors contributes to splicing decisions in different cells/tissues and pathological conditions.^[^ ^8^, ^9^, ^16^, ^17^ ^]^ SRSF3 is a major splicing factor in AS and has critical roles in the regulation of premRNA splicing.^[^ ^12^ ^]^ In this study, we provide a novel additional mechanism by which splicing factor SRSF3 recognizes and binds its cis‐RNA elements to regulate AS. We discovered that a small protein SRSP encoded by the lncRNA *LOC90024* regulates the recognition and binding of SRSF3 to exon 3 of transcription factor *Sp4*, resulting in the inclusion of *Sp4* exon 3. In cancer cells, SRSP is upregulated and increases the recognition and binding of SRSF3 on exon 3 of *Sp4* to induce the inclusion of exon 3 to produce the “cancerous” splicing variant L‐Sp4 of the *Sp4* gene; in normal cells, SRSP is absent or expressed at a low level so that SRSF3 weakly recognizes and binds to exon 3 of *Sp4*, thereby resulting in the skipping of exon 3 to produce the “noncancerous” splicing variant S‐Sp4 of the *Sp4* gene (Figure 8G).

Transcription regulation is one of the most important ways that gene expression is regulated and is mainly controlled by TFs. It has been estimated that TFs account for 20% of oncogenes in cancer.^[^ ^3^, ^4^, ^5^ ^]^ The classic examples include p53, Ets, AP‐1, c‐Myc, and NFκB. Alternations of TF activity underlie many human diseases including cancer. TF activity is altered in numerous cancer types in various ways, including by gene mutation (e.g., p53), deletion and amplification (e.g., c‐Myc), chromosomal translocation (e.g., MLL‐ELL), alteration of expression (e.g., TAL1), modifications such as phosphorylation (AP‐1), and nuclear sublocalization (NFκB).^[^ ^4^ ^]^ In addition to these ways, we show here that TF activity is also controlled by AS. SRSP, through SRSF3, induces the inclusion of exon 3 of *Sp4* to produce the “cancerous” L‐Sp4 isoform containing the transactivation domain in cancer cells; in the absence of SRSP, exon 3 of *Sp4* is skipped in normal cells to produce the S‐Sp4 isoform without the transactivation domain and oncogenic features. Therefore, our findings provide a novel additional regulatory mechanism of TF activity in cancer: AS regulation. We propose that cancer‐specific mRNA transcripts, which are produced by AS, may result in either the activation of oncogenes or the inactivation of tumor suppressors, eventually leading to tumorigenesis and cancer progression.

Previous studies showed that Sp4 is overexpressed in pancreatic, bladder, esophageal, breast, prostate, lung, colon, epidermal, thyroid, and rhabdomyosarcoma cancer cell lines and multiple myeloma cell lines, and KD of *Sp4* significantly decreases cell growth and migration and induces apoptosis in nine cancer cell lines derived from six different tumors,^[^ ^18^, ^19^ ^]^ suggesting that *Sp4* is a pro‐oncogene. However, the different *Sp4* splicing variants were not considered in these studies; only L‐Sp4 splicing variants were considered. We found for the first time that *Sp4* premRNA produces two splicing variants: L‐Sp4 and S‐Sp4. We identified a novel splicing variant, S‐Sp4, which was not previously annotated in the National Center for Biotechnology Information (NCBI) database. Similar to previous reports,^[^ ^18^ ^]^ the L‐Sp4 levels were upregulated in highly metastatic colorectal, ovarian, nasopharyngeal, and breast cancer cell sublines and primary cancer tissues compared with their parental cell lines and their matched nontumoral tissues. However, we further showed that the S‐Sp4 levels were downregulated. The total *Sp4* mRNA levels did not change. Therefore, our findings indicate that the overexpression of L‐*Sp4* in cancers reported in previous studies was not induced at the transcriptional level instead of at the posttranscriptional splicing level. Furthermore, our findings show that only L‐Sp4 is an oncogene, while S‐Sp4 has no oncogenic functions. Whether other transcription factors exert pathogenic function through disease‐specific RNA splicing is valuable to be investigated in the future.

*LOC90024* was previously annotated as a lncRNA in the NCBI database and other lncRNA databases. Its function has never been characterized. Accumulating evidence has shown that dysregulated lncRNAs, as RNA molecules, contribute to various cancer hallmarks.^[^ ^11^ ^]^ We found that lncRNA *LOC90024* actually encodes a 130‐aa small protein, which we named SRSP. *LOC90024*‐encoded small protein SRSP, but not *LOC90024* lncRNA itself, exerts oncogenic functions. Approximately 28 000 lncRNAs have been verified in the human genome, and whether there are more “hidden” proteins/peptides harbored in lncRNAs will be valuable to explore in the next step. We found that *LOC90024*‐encoded small protein SRSP may serve as an independent prognostic factor for patients with CRC. Inhibition of SRSP significantly suppresses CRC tumorigenesis. Therefore, the “hidden” proteins/peptides harbored in lncRNAs may represent promising potential biomarkers, anticancer drug targets or/and peptide drugs.

In summary, a previously annotated lncRNA *LOC90024* actually encodes a small protein SRSP. SRSP, not *LOC90024* lncRNA itself, promotes CRC tumorigenesis by interacting with SRSF3 to regulate *Sp4* premRNA splicing and produce the “cancerous” L‐Sp4 isoform. SRSP may be a potential prognostic biomarker and therapeutic target for patients with CRC.

## 4 Experimental Section

##### Cell Culture

CRC cell lines SW480, SW620, and HCT‐116, breast cancer cell line MBA‐MD‐231, ovarian cancer cell lines SK‐OV‐3 and OVAR‐3, cervix adenocarcinoma cell line HeLa, and embryonic kidney cell line HEK293T were obtained from the American Type Culture Collection and cultured under standard conditions. HCT‐116^high^, MDA‐MB‐231^high^, OVCAR‐3^high^, and SK‐OV‐3^high^ cell sublines with high metastatic abilities were previously established by the group.^[^ ^8^, ^9^ ^]^ S18 and S26 nasopharyngeal cancer cell sublines were kindly provided by Prof. Tiebang Kang at State Key Laboratory of Oncology in South China Cancer Center, Sun Yat‐sen University. Cells were regularly monitored for mycoplasma contamination.

##### Tissue Samples

Fresh frozen primary CRC tissues and their matched adjacent nontumoral colorectal tissues, which had been pathologically verified, were collected from patients with CRC at the Second Affiliated Hospital of Guangzhou Medical University and Sun Yat‐sen University Cancer Center. These cancer patients were not preoperatively treated for cancer. Informed consent was obtained from each patient, and the collection of these tissue samples was approved by the Internal Review and Ethics Boards at the Third Affiliated Hospital of Guangzhou Medicine University.

Tissue microarray chips containing CRC tissues from 101 patients with CRC and adjacent nontumoral colorectal tissues from 79 patients with CRC (including 79 pairs of matched CRC and nontumoral tissues) were purchased from Shanghai OUTDO Biotech Co., Ltd. (Shanghai), and the related clinicopathological and survival information was also provided.

##### Purification of Ribosome‐Bound RNA

The ribosome‐bound RNA was purified as previously described.^[^ ^20^ ^]^ Briefly, cells were treated with 100 µg mL^−1^ cycloheximide for 15 min and then washed twice with ice‐cold PBS buffer. These cells were treated in ice‐cold lysis buffer (1% Triton X‐100 v/v in buffer A (20 × 10^−3^ m 4‐(2‐hydroxyethyl)‐1‐piperazineethanesulfonic acid‐KOH (HEPES‐KOH) (pH 7.4), 15 × 10^−3^ m MgCl_2_, 200 × 10^−3^ m KCl, 100 µg mL^−1^ cycloheximide, and 2 × 10^−3^ m dithiothreitol (DTT))). Cell lysates were centrifuged to remove cell debris at 16 200 g for 10 min at 4 °C. The ribosome‐bound RNAs were harvested in sucrose buffer (30% sucrose in buffer A) through ultracentrifugation at 185 000 g for 5 h at 4 °C.

##### Ribosome‐Bound RNA‐seq

Ribosome‐bound RNA including mRNA and lncRNA was sequenced by Novogene Company (Beijing). The ribosome‐bound RNA was harvested using Trizol reagent. Epicentre Ribo‐zero rRNA Removal Kit (Epicentre, USA) was used to remove rRNA and rRNA free residue was cleaned up by ethanol precipitation. Sequencing libraries were generated using the rRNA‐depleted RNA by NEBNext Ultra Directional RNA Library Prep Kit for Illumina (NEB, USA) according to manufacturer's instructions. To preferentially obtain the cDNA fragments with 150–200 bp, the library fragments were purified with AMPure XP system (Beckman Coulter, Beverly, USA). The purified libraries were validated by the Agilent Bioanalyzer 2100 system. The clustering of the index‐coded samples was performed on a cBot Cluster Generation System using TruSeq PE Cluster Kit v3‐cBot‐HS (Illumia) according to the manufacturer's instructions. And then the libraries were sequenced on an Illumina Hiseq 4000 platform and 150 bp paired‐end reads were generated. Ribosome‐bound RNA‐seq data have been deposited into the Gene Expression Omnibus (GEO) under the accession number GSE139407.

##### RNA‐Seq

RNA‐seq was performed by Novogene Company (Beijing). Total RNA were harvested using Trizol reagent. RNA libraries were prepared for sequencing using standard Illumina protocols. All samples were sequenced by Illumina Novaseq6000 with a paired‐end 150 bp read length. Raw RNA‐seq reads were mapped to the human reference genome GRCh38 (hg38). The Illumina Casava1.7 program was used for base calling. Data filtering was performed with the following protocol: 1) remove reads with adaptor sequences, 2) remove reads in which the percentage of unknown bases (N) is more than 10%, and 3) remove low quality reads in which the base with a quality value ≤5 is more than 50% in a read. The mismatches ≤2 were allowed in the alignment. The expression levels were quantified using fragments per kilobase of exon per million fragments mapped method. Alternative splicing events were analyzed using the rMATS program. RNA‐seq data have been deposited into the GEO under the accession number GSE139406.

##### Generation of Anti‐SRSP Antibody

The anti‐SRSP antibody was produced by GL Biochem (Shanghai), Ltd. In brief, a Keyhole limpet hemocyanin (KLH)‐coupled epitope peptide, MRTKPQRPRATR‐Cys, was synthesized and a polyclonal antibody against SRSP was obtained from the inoculated rabbits. This anti‐SRSP antibody was further purified by affinity chromatography on columns containing the KLH‐coupled peptide.

##### IHC Detection

IHC detection was performed on CRC tissue microarray chips as previously described using the anti‐SRSP antibody.^[^ ^21^ ^]^ All IHC data were determined by two independent pathologists blinded to both the sample origins and the subject outcomes. SRSP protein level scores were evaluated using a previously described semiquantitative German scoring system including the percentage of positive cells and the staining intensity. Scores ≤4 indicated low SRSP levels (low) in CRC tissues, while scores >4 indicated high SRSP levels (high) in CRC tissues.

##### Generation of *LOC 90024* KO Cell Lines by CRISPR‐Cas9

Knockout of the gene was performed by using CRISPR‐Cas9 as previously described.^[^ ^22^ ^]^ The *LOC90024* Cas9/sgRNAs vector pGE‐4 (pU6‐gRNA1 Cas9‐puroU6‐gRNA2) was constructed by GenePharma (Shanghai, China). The two sgRNA targeting sites in *LOC90024* were 5′‐CGTAGAGGCGGCAGGCCCGC‐3′ (site 1) and 5′‐TGGTAGAGCCCTTCTCCTCC ‐3′ (site 2). The vector was transfected into HeLa cells by using Lipo2000 (Invitrogen). These cells were selected with puromycin for 36 h 24 h after transfection. Then, these cells were diluted and seeded onto 96‐well plates for single‐cell culture to obtain single cell‐derived colonies and confirmed by PCR and sequencing. The *LOC90024* KO cell colonies were selected, cultured, and saved for subsequent use.

##### Mouse Models for Tumor Growth and Metastasis

The in vivo tumor growth investigation was performed as previously described.^[^ ^22^ ^]^ Male BALB/c nude mice (3–4 weeks old) were purchased from Charles River Laboratories in China (Beijing). Briefly, the indicated CRC cells (1 × 10^6^) were injected subcutaneously into the right or left armpits of each mouse to establish a CRC xenograft model (*n* = 6). After 23 d, these mice were euthanized and the tumor xenografts were excised and weighed.

The in vivo metastasis analysis was performed as previously described.^[^ ^22^ ^]^ Male NOD‐SCID mice (3–4 weeks old) were obtained from Charles River Laboratories in China (Beijing). Briefly, the indicated CRC cells (2 × 10^6^) were injected into the blood of each mouse through the tail vein (*n* = 5). Each mouse was injected with d‐Luciferin potassium salt through the abdominal cavity 60 d after implantation. The metastatic foci were visualized by an IVIS 200 Imaging System (Xenogen).

The mice used in this study were bred and maintained under defined conditions at the Animal Experiment Center of the College of Medicine (SPF grade), Jinan University. The animal experiments were approved by the Laboratory Animal Ethics Committee of The Third Affiliated Hospital of Guangzhou Medicine University and Jinan University and conformed to the legal mandates and national guidelines for the care and maintenance of laboratory animals.

##### Mass Spectrometry and Protein Identification

Protein identification was performed using a mass spectrometer as previously described.^[^ ^21^ ^]^ Briefly, the digested peptide mixtures were analyzed using a nano‐LC‐MS/MS mass spectrometer (AB SCIEX TripleTOF 5600, USA). The WIFF RAW files were converted into peaklist files through Protein Pilot Software v4.5 (AB SCIEX). Proteins were identified using the Mascot (v2.3.02) program against the generated SRSP protein database with the default parameters. For the identification of SRSP‐interacting proteins, proteins with FDR ≤1% and unique peptides ≥2 were selected.

##### Protein‐RNA Binding Assay

The 5′‐biotin‐labeled RNAs of exon 3 of *Sp4* containing the SRSF3‐bound sites were synthesized: E3(848–866): CACAACCACUGCUUCAAC; E3(848–866)mut in which the SRSF3‐bound site CAACCA was mutated to GUUGGU: CAGUUGGUCUGCUUCAAC; and negative control RNA of 1–20 nt of exon 3 of *Sp4* E3(1–20): GACUCUCAGCCCUCUCCUCU. Protein‐RNA binding analysis was performed as previously described.^[^ ^16^ ^]^ In brief, cellular nuclear proteins were obtained using the Nuclear and Cytoplasmic Protein Extraction Kit (Beyotime). Each 1 nmol biotin‐labeled RNA was bound with 100 µL streptavidin‐agarose beads (Sigma) overnight at 4 °C. RNA‐immobilized beads were then incubated with the cellular nuclear proteins at 30 °C. These beads were washed twice with washing buffer and then eluted by adding protein loading buffer. The eluted mixtures were detected by Western blotting using anti‐SRSF3, ‐SRSP, or ‐HA antibodies.

##### Detection of *Sp4* Splicing

The forward and reverse primers used for detecting the two *Sp4* splicing variants were matched to exon 2 and exon 4 of *Sp4* premRNA, respectively. Total RNA was prepared using TRIzol. cDNAs were synthesized by using the PrimeScript RT Reagent Kit with gDNA Eraser (TaKaRa). *Sp4* splicing variants were detected by RT‐PCR. The primers were provided in Table S5 in the Supporting Information.

##### Statistical Analyses

Prism 8 and SPSS 16.0 software were used for statistical analyses. Significances between two groups were analyzed using two‐tailed unpaired Student's *t*‐tests or Mann–Whitney *U*‐tests. The significance of the growth curves was analyzed by two‐way ANOVA. Survival curves were determined using the Kaplan–Meier method with the log‐rank test. The data are presented as the mean ± SD except where stated otherwise. The differences with *p < 0.05, **p < 0.01, or ***p < 0.001 were considered statistically significant.

##### Data Availability

The ribosome‐bound RNA‐seq and RNA‐seq data obtained in this study have been uploaded to NCBI GEO datasets under accession numbers GSE139407 and GSE139406, respectively.

## Conflict of Interest

The authors declare no conflict of interest.

## Supporting information

Supporting Information

Supplemental Table 1
