# Supplementary material for: Small Protein Hidden in lncRNA LOC90024 Promotes “Cancerous” RNA Splicing and Tumorigenesis
Source: Adv Sci (Weinh). 2020 Mar 11;7(10):1903233. doi: 10.1002/advs.201903233 (PMC7237858; doi:10.1002/advs.201903233)
Supplement: Supplementary file 1 — Supporting Information [file ADVS-7-1903233-s001.pdf]

Copyright WILEY-VCH Verlag GmbH & Co. KGaA, 69469 Weinheim, Germany, 2020.

ADVANCED SCIENCE
Open Access

Supporting Information

for *Adv. Sci.,* DOI: 10.1002/advs.201903233

Small Protein Hidden in lncRNA *LOC90024* Promotes "Cancerous" RNA Splicing and Tumorigenesis

*Nan Meng, Min Chen, De Chen, Xin-Hui Chen, Ji-Zhong Wang, Song Zhu, Yu-Tian He, Xiao-Lan Zhang, Rui-Xun Lu, and Guang-Rong Yan**

Supporting Information

Small Protein Hidden in lncRNA *LOC90024* Promotes "Cancerous" RNA Splicing and Tumorigenesis

Nan Meng, Min Chen, De Chen, Xin-Hui Chen, Ji-Zhong Wang, Song Zhu, Yu-Tian He, Xiao-Lan Zhang, Rui-Xun Lu, Guang-Rong Yan*

Supporting Figures

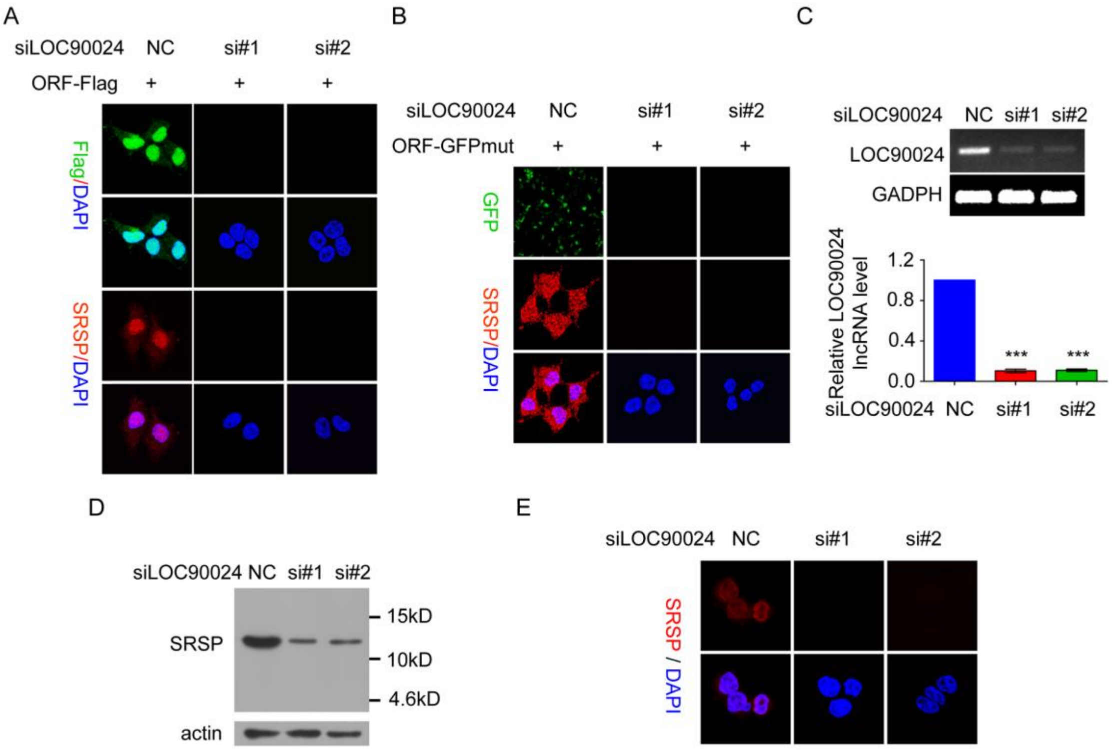

Figure S1. Our anti-SRSP antibody specifically detects SRSP. (A) The *LOC90024* ORF-Flag construct and anti-*LOC90024* siRNA were cotransfected into HCT-116 cells; immunostaining of SRSP-Flag fusion protein was performed with anti-Flag and anti-SRSP antibodies. (B) The *LOC90024* ORF-GFP construct and anti-*LOC90024* siRNA were cotransfected into HCT-116 cells; SRSP-GFP was directly visualized by fluorescence or analyzed by immunostaining with anti-SRSP antibody. (C-E) Endogenous *LOC90024* expression in HCT-116 cells was silenced, and the *LOC90024* lncRNA level (C), SRSP level (D), and SRSP-immunostaining (E) were analyzed. Data are represented as mean ± SD. ***$p<0.001$.

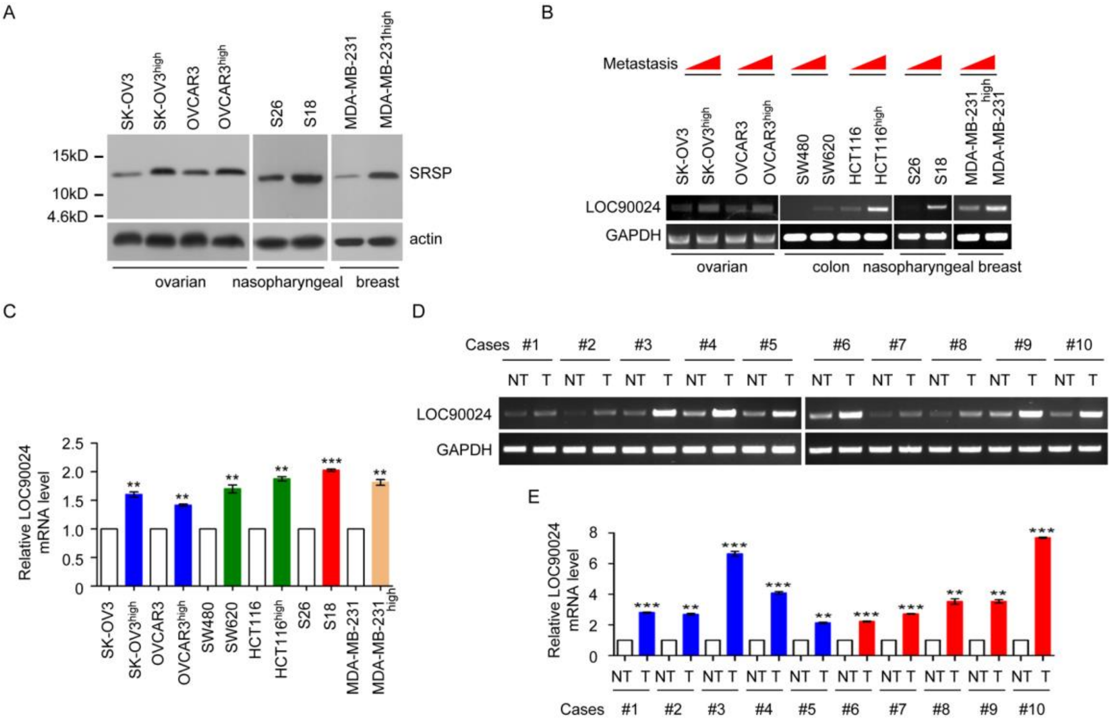

Figure S2. *LOC90024* and its encoded-SRSP levels were upregulated in high-metastatic cancer cell sublines and primary tumor tissues compared with their matched low-metastatic parental cell lines and adjacent nontumoral tissues, respectively. (A) The SRSP levels were determined in the indicated high-metastatic cancer cell sublines and their matched low-metastatic parental cell lines. (B, C) The *LOC90024* lncRNA levels were determined between the indicated high-metastatic cancer cell sublines and their matched low-metastatic parental cell lines by RT-PCR (B) and qPCR (C) (n=3). (D, E) The *LOC90024* lncRNA levels were determined in ten pairs of primary tumor tissues and their matched adjacent nontumoral tissues by RT-PCR (D) and qPCR (E). Data are represented as mean ± SD. **p<0.01 or ***p<0.001.

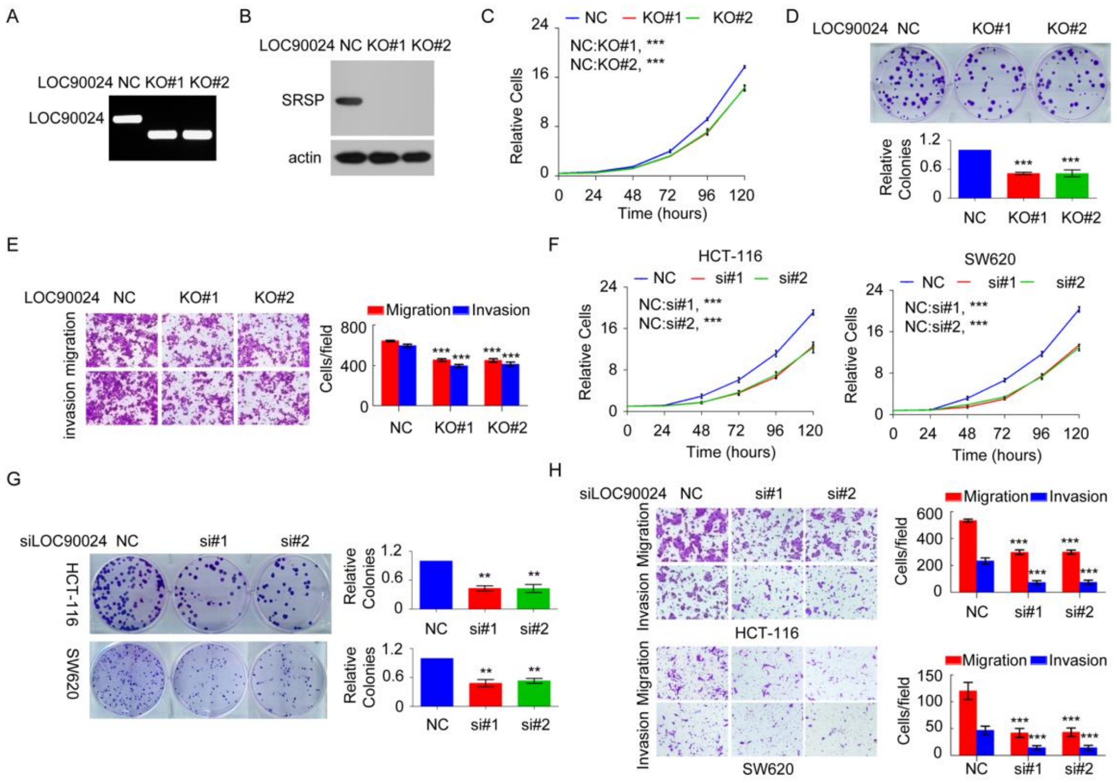

Figure S3. KO or KD of *LOC90024* inhibits cancer cell growth, colony formation, migration and invasion. (A) *LOC90024* was knocked out in HeLa cells by CRISPR-Cas9. (B-E) SRSP level (B), cell growth (C), colony formation (D), migration and invasion (E) were determined in two *LOC90024* KO HeLa cell clones (n=3). (F-H) *LOC90024* expression in HCT-116 and SW620 CRC cells was knocked down by two anti-*LOC90024* siRNAs, and cell growth (C), colony formation (D), migration and invasion (E) were determined (n=3). Data are represented as mean ± SD. **p<0.01 or ***p<0.001.

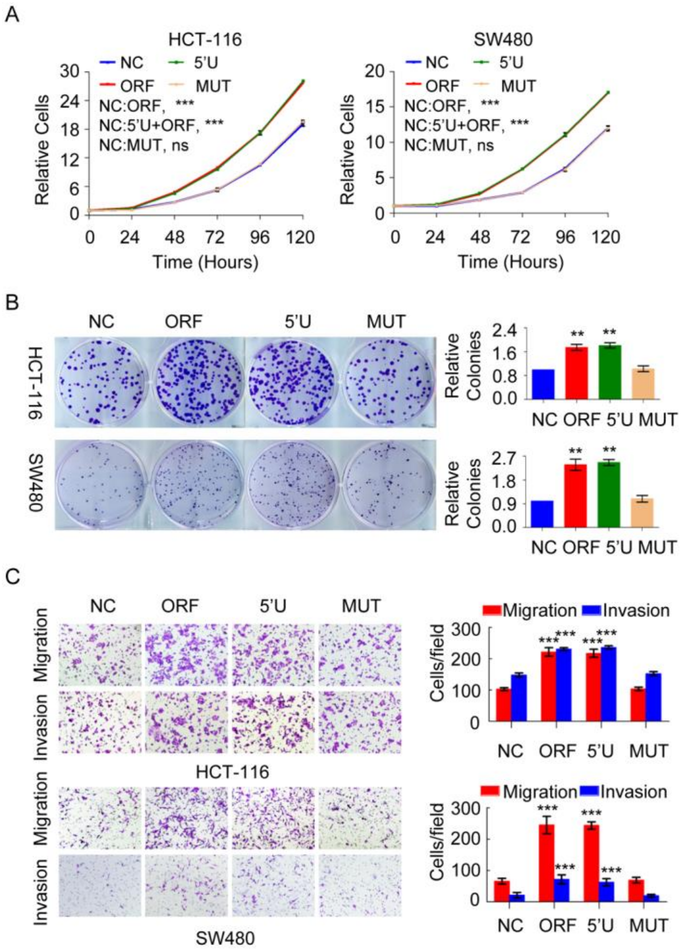

Figure S4. SRSP, not *LOC90024* lncRNA itself, promotes CRC cell growth, colony formation, migration and invasion. The indicated *LOC90024* constructs were transfected into two CRC HCT-116 and SW480 cells, and cell growth (C), colony formation (D), migration and invasion (E) were determined (n=3). Data are represented as mean ± SD. **p<0.01 or ***p<0.001.

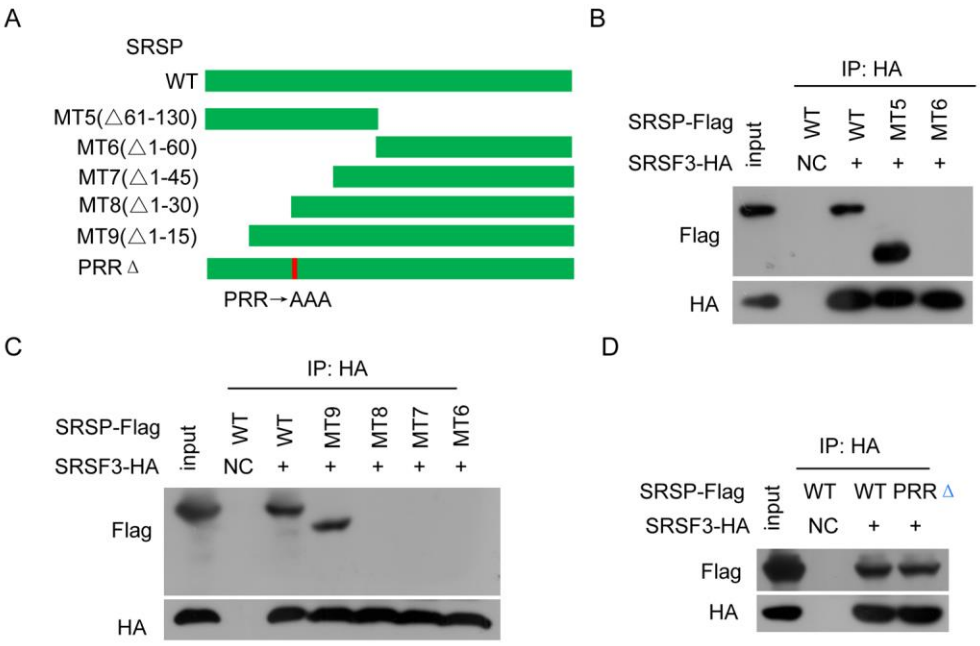

Figure S5. The 15-30 aa sequence of SRSP interacts with SRSF3. (A) Schematic diagram of wild-type *SRSP* and its mutant constructs. (B-D) The indicated *SRSP-Flag* mutants with *SRSF3-HA* vector were cotransfected into HEK293T cells, the SRSF3-HA complexes were co-IPed using anti-HA antibody, and the SRSP-Flag mutant was detected using anti-Flag antibody.

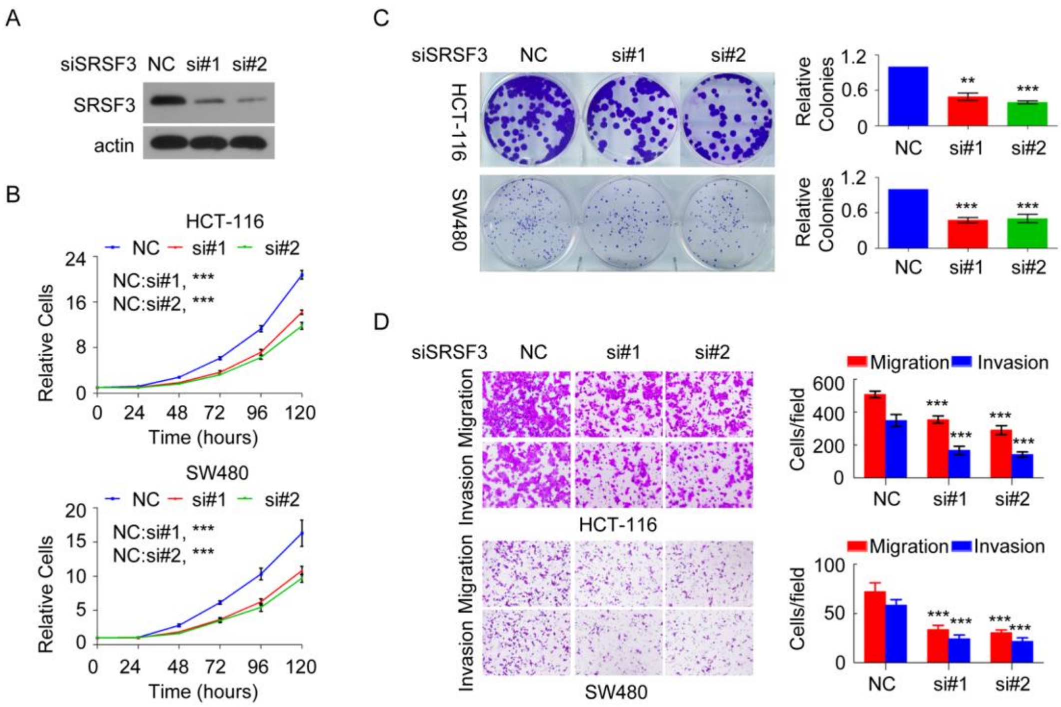

Figure S6. KD of *SRSF3* suppresses CRC cell growth, colony formation, migration and invasion, phenocopying the oncogenic functions of KD of *SRSP*. Two anti-*SRSF3* siRNAs were transfected into two CRC cell lines, HCT-116 and SW480, and SRSF3 level (A), cell growth (B), colony formation (C), migration and invasion (D) were analyzed (n=3). Data are represented as mean ± SD. **p<0.01 or ***p<0.001.

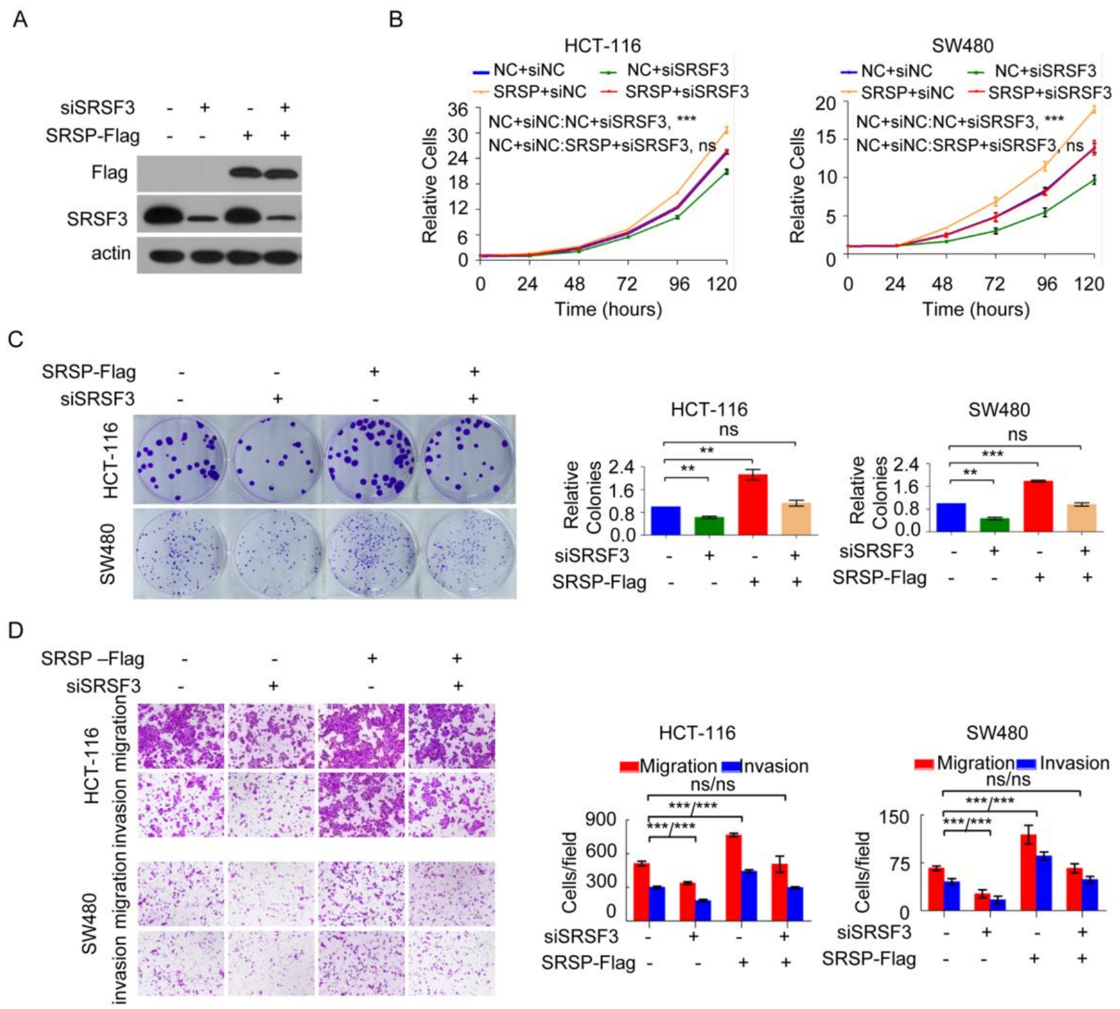

Figure S7. SRSP promotes CRC cell growth, colony formation, migration and invasion through SRSF3. HCT-116 and SW480 CRC cells were cotransfected with *SRSP-Flag* vector together with anti-*SRSF3* siRNA, and SRSP-Flag and SRSF3 levels (A), cell growth (B), colony formation (C), migration and invasion (D) were determined (n=3). Data are represented as mean ± SD. **p<0.01 or ***p<0.001.

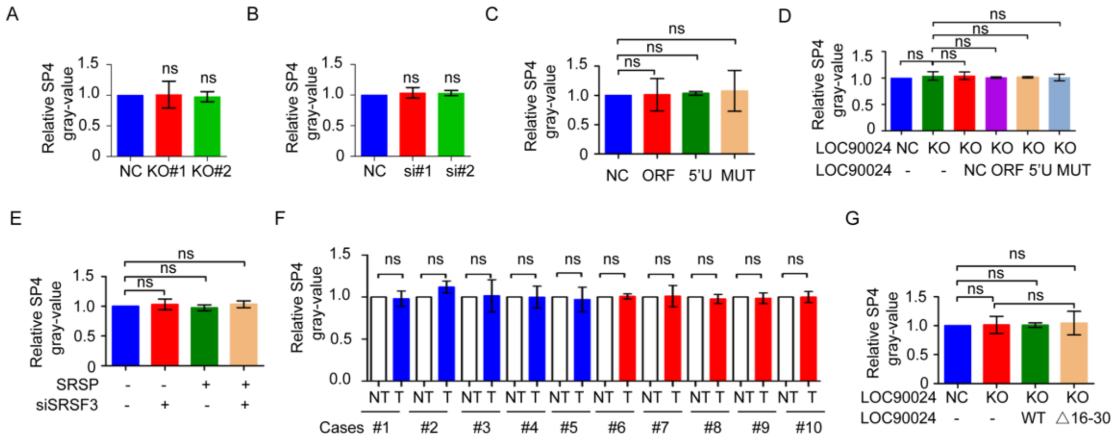

Figure S8. SRSP did not change the total *Sp4* mRNA level. (A-F) Total *Sp4* mRNA levels were analyzed in Figure 6A-6F. (G) Total *Sp4* mRNA levels were analyzed in Figure 8C. Data are represented as mean ± SD. ns indicates no significance.

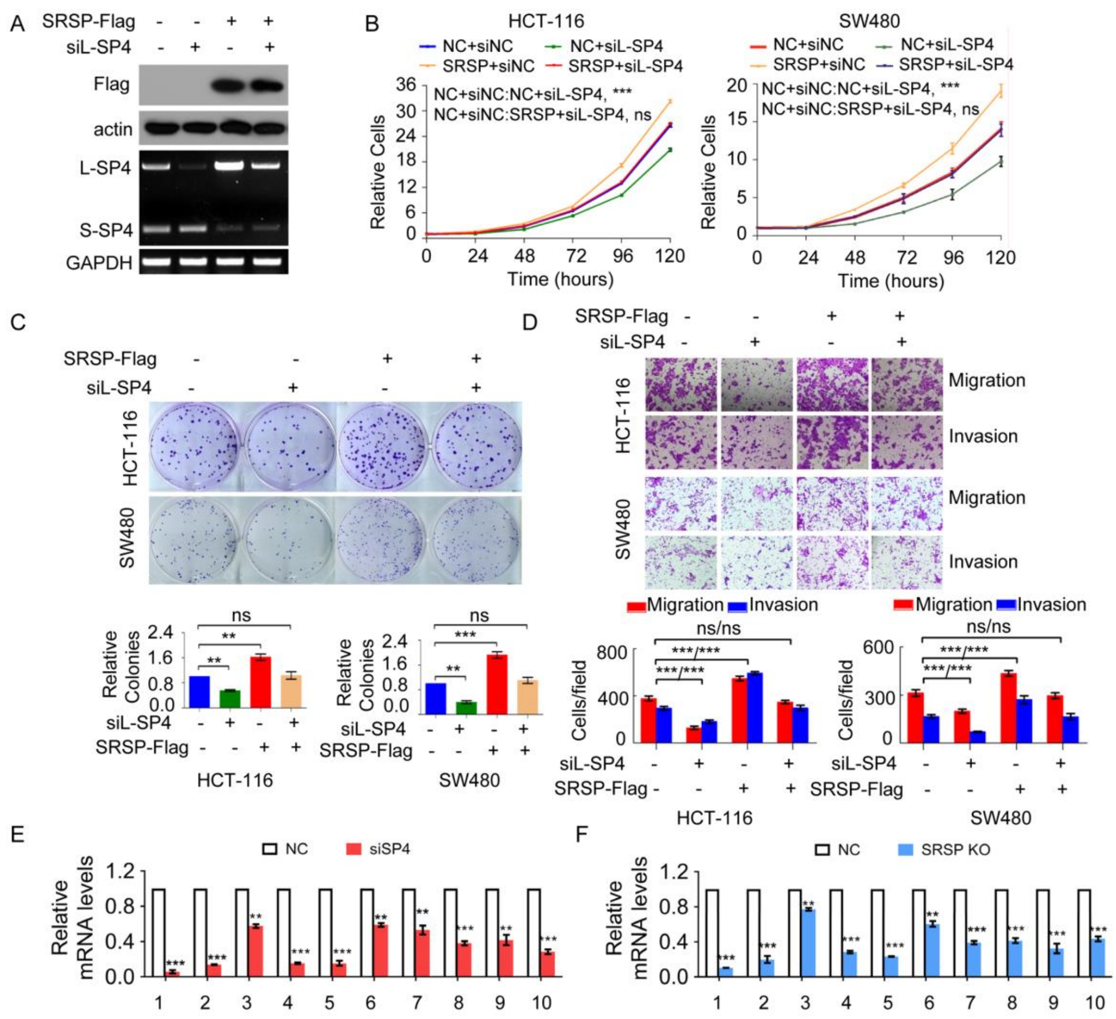

Figure S9. SRSP promotes CRC cell growth, colony formation, migration and invasion mainly through the L-SP4 isoform. HCT-116 and SW480 CRC cells were cotransfected with *SRSP-Flag* vector together with anti-*L-SP4* siRNA, and SRSP-Flag, L-SP4 and S-SP4 levels (A), cell growth (B), colony formation (C), and migration and invasion (D) were determined (n=3). (E) HeLa cells were transfected with anti-SP4 siRNA, the mRNA levels of the indicated genes were determined using qRT-PCR (n=3). (F) The mRNA levels of the indicated genes were determined in LOC90024 KO HeLa cells using qRT-PCR (n=3). Genes in (E, F): 1, PRKAG2; 2, ZNF24; 3, COMP; 4, COMMD2; 5, TMEM159; 6, TOP1MT; 7: IRF6; 8, STXBP2; 9, CITED4; 10, CDH15. Data are represented as mean ± SD. **p<0.01 or ***p<0.001.

Supporting Tables S1. Correlations between SRSP level and clinicopathological features in 101 CRC cases.

Clinical characters	All cases	SRSP		*P*-value[a)]
		Low	High	
Sex				0.907
Female	44	16	28	
Male	56	21	35	
Age (Years)				
<60	21	10	11	0.126
≥60	74	22	52	
Tumor size (cm)				0.349
<5	38	16	22	
≥5	61	20	41	
Histological Grade				0.001
G1-G2	63	31	32	
G3	38	6	32	
pT status				0.122
1-2	6	4	2	
3-4	91	32	59	
pN status				0.026
0	61	28	33	
1-2	38	9	29	
Clinical Stage				0.004
I-IIA	54	27	27	
IIB-IV	46	10	36	

[a)] Pearson Chi-square test.

Supporting Table S2. Univariate and multivariate analysis of different prognostic parameters, including SRSP, in 101 patients with CRC.

Clinical character	OS(months)			
	Univariate analysis		Multivariatie analysis	
	HR(95% CI)	*P* value[a)]	HR(95% CI)	*P* value[a)]
Sex (Female vs. Male)	1.038 （0.619-1.740）	0.888	1.298 (0.698-2.416)	0.410
Age (＜60 vs. ≥60)	1.836 （0.901-3.740）	0.094	1.320 (0.606-2.874)	0.484
Tumor size (＜5cm vs.≥5cm)	1.201 （0.708-2.037）	0.497	1.302 (0.726-2.337)	0.376
Histological Grade (G1-G2 vs.G3-G4)	3.673 （2.182-6.184）	0.0001	1.962 (1.083-3.556)	0.026
pT status (1-2 vs.3-4)	2.413 （0.588-9.897）	0.221	0.855 (0.193-3.798)	0.837
pN status (0 vs. 1-2)	2.556 （1.519-4.302）	0.0001	2.545 (1.369-4.729)	0.003
SRSP (Low vs. High)	9.905 （4.232-23.180）	0.0001	7.692 (2.982-19.841)	0.0001

[a)]Cox proportional hazard model.

Supporting Table S4. The siRNA sequences used in this study.

Gene name	siRNA No.	siRNA sequences
LOC90024	si#1	Sense: 5’-GAGGAGACGGGAGAAACAUTT-3’<br>Anti-sense: 5’-AUGUUUCUCCGUCUCCUCTT-3’
	si#2	Sense: 5’-GGUCGGAAGGAAAGUAGAGTT-3’<br>Anti-sense: 5’-CUCUACUUUCCUUCCGACCTT-3’
SRSF3	si#1	Sense: 5’-CAUCGUGAUUCCUGUCCAUTT-3’<br>Anti-sense: 5’-AUGGACAGGAAUCACGAUGTT-3’
	si#2	Sense: 5’-CCCUCGAGAUGAUUAUCGUTT-3’<br>Anti-sense: 5’-ACGAUAAUCAUCUCGAGGGTT-3’
Sp4	si#1	Sense: 5’-GGUGCAAAUUGUAGGCCAATT-3’<br>Anti-sense: 5’-UUGGCCUACAAUUUGCACCTT-3’
	si#2	Sense: 5’-GCUCCAACUUUAACACCUUTT-3’<br>Anti-sense: 5’-AAGGUGUUAAAGUUGGAGCTT-3’
	si#3	Sense: 5’-CCAGCAGACUUCUGAUCAATT-3’<br>Anti-sense: 5’-UUGAUCAGAAGUCUGCUGGTT-3’
NC		Sense:<br>5‘-GCACAAGCUGGAGUACAACUACATT-3‘<br>Anti-sense:<br>5’-UGUAGUUGUACUCCAGCUUGUGCTT-3’

Supporting Table S5. The RT-PCR or qPCR primers used in this study.

Gene name	Primers
RT-PCR or PCR	
LOC90024	Forward: 5'-GTGCCTTGTCGGCTGGGTTA-3'<br>Reverse: 5'-CCTTCTGGTAGACGGAGACCC-3'
Sp4	Forward: 5'-ACCTCTGAGCCAGAGAATAAC-3'<br>Reverse: 5'-CCTTCTCCTTCCCTACAATTAG-3'
GAPDH	Forward: 5'-CGGAGTCAACGGATTTGGTCGTAT-3'<br>Reverse: 5'-AGCCTTCTCCATGGTGGTGAAGAC-3'
LOC90024 (CRISPR-Cas9, gDNA, PCR)	Forward: 5'- TTTTCTTCCAGCTGCCGGACCA-3'<br>Reverse: 5'- CCCTCTTGTGGGCCACGCAC-3'
qRT-PCR	
LOC90024	Forward: 5'-GCGGACTAGCGGAGGAGGAT-3'<br>Reverse: 5'-GGGTGTAGAATGACTGGAAGGAGC-3'
PRKAG2	Forward: 5'-TATGGGAAGCGCGGTTATGGA-3'<br>Reverse: 5'-AGGAGCGGCATGGCGAAGGA-3'
ZNF24	Forward: 5'-GCGAAGAGGGATCAA-3'<br>Reverse: 5'-AACCCAAGTCTGTAGCTC-3'
COMP	Forward: 5'-CCGACACCGCCTGCGTTCTT-3'<br>Reverse: 5'-CAGCGCCGCGTTGGTTT-3'
COMMD2	Forward: 5'-CTGCCGGGTGAAATCGTAGG-3'<br>Reverse: 5'-GGTTTGCGCCGCGTCTC-3'
TMEM159	Forward: 5'-TGGGCTTCGTATCACTC-3'<br>Reverse: 5'-CTGGCAGAAAGTCACAACTG-3'
TOP1MT	Forward: 5'-GCACAAGGGCCCGTACTTC-3'<br>Reverse: 5'-TCCTCCGCTGCCACGCTCAA-3'
STXBP2	Forward: 5'-CCACGGAGAAGTCGGTTCAG-3'<br>Reverse: 5'-CCACCTTTGCCAGACGAGAG-3'

CITED4	Forward: 5’-CGGGAGGACAGTTTGGCTTC-3’
	Reverse: 5’-GGGAGAGGACACGATCCAAG-3’
IRF6	Forward: 5’-AGCGGTCAAGGGAAAGACAA-3’
	Reverse: 5’-CCAGATGAGCCCAGGGTAGA-3’
CDH15	Forward: 5’-GAGAACCCACTTCGGACCAG-3’
	Reverse: 5’-TCTTCCGGGTCGTAGTCCTT-3’
GAPDH	Forward: 5’-GAAGGTGAAGGTCGGAGTC-3’
	Reverse: 5’-AAGATGGTGATGGGATTTC-3’

Supporting experimental section

Plasmid construction

The lncRNA *LOC90024* ORF and 5'UTR-ORF sequences were cloned into the *eGFP* vector in which the start codon ATGGTG was mutated to ATTGTT. The lncRNA *LOC90024* ORF and 5'UTR-ORF, *SRSF3* ORF, *L-Sp4* and *S-Sp4* ORF sequences, and the indicated sequences of *SRSP* or *SRSF3* mutants, which were fused with *Flag* or *HA* tag sequences, were cloned into the pcDNA3.1 (+) vector by the ClonExpress II One Step Cloning Kit or the Mut Express II Fast Mutagenesis Kit v2 (Vazyme, China).

Construct of synonymous *L-Sp4* mutant

The anti-*Sp4* siRNA#3 (sense: CCAGCAGACUUCUGAUCAATT, antisense: UUGAUCAGAAGUCUGCUGGTT) targeted the *L-Sp4* splicing variant but not the *S-Sp4* splicing variant. The anti-*Sp4* siRNA#3-targeted sequence in *L-Sp4-Flag vector,* CCAGCAGACTTCTGATCAA, was synonymously mutated to TCAACAAACATCAGACCAG. The synonymous mutant s*L-Sp4* vector was generated.

RNA interference

SiRNA against *LOC90024*, *SRSF3* and Sp4 genes and corresponding scrambled siRNA (GenePharma) were transfected into the cells with RNAiMAX (Invitrogen). The plasmids together with siRNAs were co-transfected using Lipofectamine 2000 (Invitrogen). The siRNA sequences are provided in Supporting Table S4.

Immunostaining assay

Cells were transfected with *LOC90024* ORF-, 5'UTR-ORF-, or 5'UTR-ORFmut-fused *GFP* vector, and *GFP* fluorescence was directly visualized and recorded. Cells were transfected with the indicated vectors or siRNAs for 24 h and then plated and cultured on glass cover slips for 24 h. These cells were fixed, permeabilized, and incubated with anti-SARP or -Flag antibodies. Alexa Fluor 488- or Cy3-conjugated secondary IgG antibodies were used for staining. DAPI was used to stain cellular nuclei. The immunofluorescence was observed by laser scanning confocal microscopy.

Western blotting

Cell and tissue lysates were separated by SDS-PAGE and then electroblotted onto a PVDF membrane. Antibodies against GFP (Cat#50430-2-AP; RRID: AB_11042881, proteintech; 1:1000), Flag (Cat#M185-3L; RRID: AB_11123930, MBL; 1:2000), HA (Cat#561; RRID: AB_591839, MBL; 1:2000), SRSP (our produced, 1:400), SRSF3 (Cat#ab198291, Abcam; 1:1500), Sp4 (Cat#PA5-80058; RRID: AB_2747173, Invitrogen; 1:2000), and β-actin (Cat#60008-1-Ig; RRID: AB_2289225, Proteintech; 1:5000) were used for detecting the indicated proteins.

RT-PCR and qRT-PCR

Total RNA was extracted using TRIzol total RNA isolation reagent (Invitrogen). *LOC90024* lncRNA and *GAPDH* mRNA levels were analyzed using RT-PCR and qRT-PCR. The primers used in this study are provided in Supporting Table S5.

Cell growth assay

Cell growth assays were performed as previously described.[1] In brief, 1×10^4 cells were plated in 96-well culture plates and cultured in culture medium supplemented with 10% FBS 12 h after transfection with the indicated siRNAs and/or plasmids. The cell number was counted at 24, 48, 72, 96, and 120 h.

Colony formation assay

Colony formation abilities were determined as previously described.[2] In brief, 250 cells were plated in 6-well culture plates and cultured in medium supplemented with 10% FBS 12 h after transfection with the indicated siRNAs and/or plasmids. Then, these cells were stained with crystal violet solution. The colony numbers were counted (n=3).

Migration and invasion

The migration and invasion abilities were determined using Transwell chambers as previously described.[3] Twelve hours after transfection with the indicated siRNAs or/and plasmids, 1×10^5 cells in 100 μL RPMI 1640 medium with 0.05% FBS were added to the upper Transwell chambers for the migration assay (8.0 μM pore size, BD) or the upper Transwell chambers coated with Matrigel for the invasion assay, and RPMI 1640 medium with 10% FBS was added to the bottom chamber. Migrated and

invaded cells were stained with 5% crystal violet and counted (n=3).

Generation of cell lines with stable expression

Our previously established HCT-116-Luc cell line stably expressing luciferase (Luc) was further infected with lentiviruses expressing the indicated *LOC90024* ORF, 5'UTR-ORF, or 5'UTR-ORFmut constructs containing *Flag* tags and selected using puromycin. HCT-116-Luc-ORF-Flag, HCT-116-Luc-5'UTR-ORF-Flag or HCT-116-Luc-5'UTR-ORFmut-Flag cell lines were established. The ectopic expression of the SRSP-Flag fusion protein in these cell lines was validated by Western blotting.

Coimmunoprecipitation (co-IP)

The SRSP-Flag or SRSF3-HA complexes were coimmunoprecipitated (co-IPed) using anti-Flag or anti-HA antibodies, respectively. The co-IPed complexes were captured on Protein A/G agarose beads (Santa Cruz). Then, the complexes were separated by SDS-PAGE. The gels were stained with silver for protein identification using mass spectrometry or used for detection with the indicated antibodies via Western blotting.

For the identification of the SRSP-bound proteins, three independent experiments were performed. The differential gel bands and their matched negative gel bands were excised, in-gel digested with trypsin. These digested peptide mixtures were identified using mass spectrometry.

References

[1] J. Z. Huang, M. Chen, Chen, X. C. Gao, S. Zhu, H. Huang, M. Hu, H. Zhu, G. R. Yan, *Mol. Cell* **2017**, 68, 171.

[2] M. Chen, X. J. Sheng, Y. Y. Qin, S. Zhu, Q. X. Wu, L. Jia, N. Meng, Y. T. He, G. R. Yan, *Theranostics* **2019**, 9, 676.

[3] J. Z. Huang, M. Chen, M. Zeng, S. H. Xu, F. Y. Zou, D. Chen, G. R. Yan, *J. Pathol.* **2016**, 239, 186.
